# Novel Use of Hypoxia-Inducible Polymerizable Protein to Augment Chemotherapy for Pancreatic Cancer

**DOI:** 10.3390/pharmaceutics14010128

**Published:** 2022-01-05

**Authors:** Andrew Gdowski, Hamed Hayatshahi, Rafal Fudala, Rohan Joshi, Jin Liu, Jamboor K. Vishwanatha, Rohan Jeyarajah, Paul Guzik, Amalendu P. Ranjan

**Affiliations:** 1 Lineberger Comprehensive Cancer Center, University of North Carolina, Chapel Hill, NC 27599, USA; andrewgdowski@gmail.com; 2 Reata Pharmaceuticals Inc., Plano, TX 75024, USA; Hamed.Hayatshahi@reatapharma.com; 3 Department of Microbiology, Immunology and Genetics, University of North Texas Health Science Center, Fort Worth, TX 76107, USA; Rafal.fudala@unthsc.edu (R.F.); Rohan.Joshi@my.unthsc.edu (R.J.); Jamboor.vishwanatha@unthsc.edu (J.K.V.); 4 Department of Pharmaceutical Sciences, University of North Texas System College of Pharmacy, University of North Texas Health Science Center, Fort Worth, TX 76107, USA; Jin.Liu@unthsc.edu; 5 TCU and UNTHSC School of Medicine, Fort Worth, TX 76107, USA; rohanjeyarajah@gmail.com; 6 Division of Gastroenterology & Hepatology, Loma Linda University Medical Center, Loma Linda, CA 92354, USA; paul.r.guzik@gmail.com

**Keywords:** hemoglobin S, pancreatic cancer, gemcitabine, polymerization, hypoxia

## Abstract

Pancreatic ductal adenocarcinoma (PDAC) is one of the most aggressive malignancies and is the fourth leading cause of cancer-related deaths in the United States. Unfortunately, 80–85% of patients are diagnosed with unresectable, advanced stage tumors. These tumors are incurable and result in a median survival less than approximately six months and an overall 5-year survival rate of less than 7%. Whilst chemotherapy is a critical treatment, cure is not possible without surgical resection. The poor clinical outcomes in PDAC can be partially attributed to its dense desmoplastic stroma, taking up roughly 80% of the tumor mass. The stroma surrounding the tumor disrupts the normal architecture of pancreatic tissue leading to poor vascularization, high intratumoral pressure along with hypoxia and an acidic tumor microenvironment. This complicated microenvironment presents a significant challenge for drug delivery. The current manuscript discusses a novel approach to overcome many of these various obstacles. A complex of gemcitabine (GEM) and hemoglobin S (HbS) was formulated, which self-polymerizes under hypoxic and acidic conditions. When polymerized, HbS has the potential to break the tumor stroma, decrease intratumoral pressure, and therefore improve the treatment efficacy of standard therapy. Intratumoral injection of HbS with a fluorescent small molecule surrogate for GEM into a pancreatic tumor xenograft resulted in improved dissemination of the small molecule throughout the pancreatic tumor. The self-polymerization of HbS + GEM was significantly more effective than either agent individually at decreasing tumor size in an in vivo PDAC mouse model. These findings would suggest a clinical benefit from delivering the complex of GEM and HbS via direct injection by endoscopic ultrasound (EUS). With such a treatment option, patients with locally advanced disease would have the potential to become surgical candidates, offering them a chance for cure.

## 1. Introduction

Pancreatic ductal adenocarcinoma (PDAC) is one of the most lethal human malignancies with an overall 5-year survival rate below 7% [1]. The majority of patients with PDAC are not candidates for surgical resection due to local tumor invasion and metastasis. Even with currently available systemic and radiation therapy options, overall survival is limited [2,3]. The antimetabolite small molecule gemcitabine (GEM) is one potential agent used to treat PDAC but results in only minimal increased survival. Despite recent advances with additional chemotherapy regimens, the efficacy of chemotherapy remains poor [4]. Increasing evidence shows that the pancreatic tumor microenvironment presents several barriers to treatment delivery. The major challenges are enriched tumor stromal components and a disorganized vasculature of pancreatic cancer tissues, which make it difficult to deliver a sufficient amount of chemotherapy into the tumor due to higher interstitial pressure [5,6]. Furthermore, insufficient tissue oxygenation, or hypoxia, contributes to solid tumor expansion [7]. Efforts have been made to develop and study drugs that target this novel pancreatic cancer microenvironment [8]. However, so far, these newer agents have been insufficient.

**Scheme 1:**
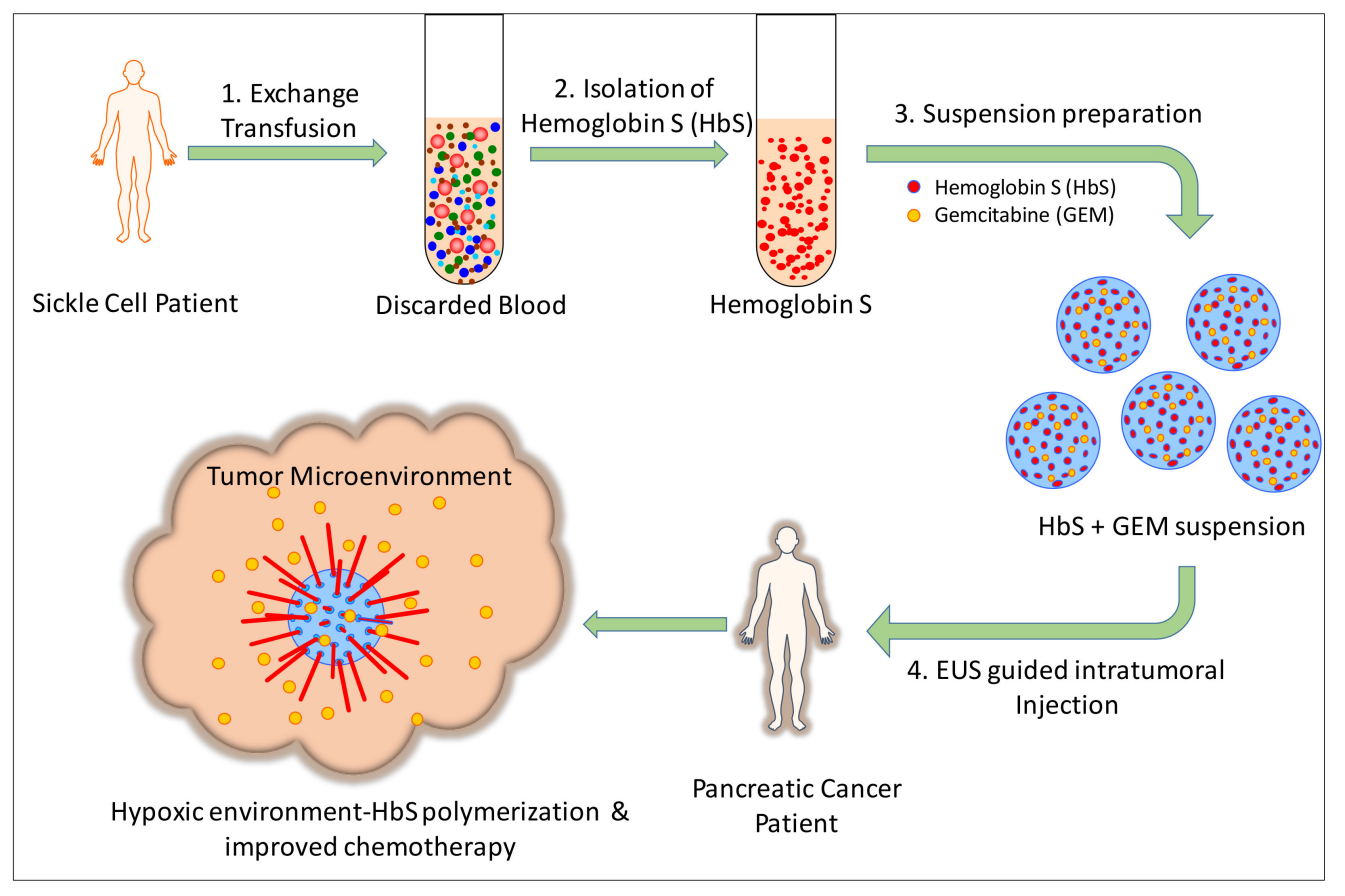
Theoretical overview of hypoxia-induced polymerization of HbS and synergistic chemotherapy. (1.) Blood could be harvested during exchange transfusion from a patient with known sickle cell anemia or HbS could be engineered in a lab. (2.) HbS could be isolated and pooled together. (3.) HbS and GEM can be complexed in solution. (4.) An endoscopic ultrasound (EUS) injection can deliver therapy directly to the tumor. Once in the tumor microenvironment, HbS polymerizes and allows improved diffusion of GEM throughout the tumor.

Sickle cell hemoglobin (HbS) is a tetrameric protein and a variant of the normal hemoglobin protein in which a point mutation causes a substitution of valine for glutamic acid on the beta globin chain of the tetrameric molecule. The consequence of this substation is that when HbS molecules are in a deoxygenated or acidic state, there is a confirmation change in the protein that facilitates noncovalent interactions between the side chain residues of *B*Val6 and the hydrophobic grooves of amino acid residues of a neighboring tetramer. This results in polymerization and precipitation of HbS, which forms the classic sickled shape of red blood cells seen in sickle cell anemia [9,10]. In this paper, we tested the hypothesis that polymerization of HbS can be exploited in the hypoxic and acidic pancreatic tumor microenvironment to overcome the dense reactive tumor stroma and utilize this strategy to simultaneously distribute a small molecule throughout the tumor to decrease tumor size. For PDAC, direct injection into the tumor using interventional endoscopic ultrasonography (EUS) guided local therapy could easily be used to deliver this therapy directly to the tumor in a clinical setting [11] (Scheme 1).

## 2. Materials and Methods

### 2.1. Materials

Gemcitabine hydrochloride (GEM·HCl) (≥98%) was purchased from Proteochem, UT, USA. Hemoglobin S (HbS) was purchased from Sigma-Aldrich, St. Louis, MO, USA. RPMI 1640 medium, fetal bovine serum (FBS), and phosphonate buffered solution (PBS) were supplied from Hyclone, Marlborough, MA, USA. Cell culture procedures: BxPC3 cells used for animal studies were purchased from American Type Culture Collection (ATCC) and tested negative for mycoplasma contamination. Cells were grown in RPMI 1640 supplemented with 2 mM L-glutamine and 10% FBS and used for in vivo studies.

### 2.2. Suspension Preparation

HbS was suspended at a concentration of 20 mg/mL in PBS. Gemcitabine hydrochloride was next added into a solution by suspending in saline separately. The gemcitabine solution was added to the HbS solution to make the final suspension of gemcitabine and HbS that was then vortexed for 30 s. For animal experiments, the solution was drawn up in 1 cc, 30 G syringes for injection. This was prepared immediately prior to use.

### 2.3. In Silico Molecular Docking of HbS and GEM

We prepared the PDB entries 1NEJ [12] and 2HBS [13] as the crystal structures of HbS in oxygenated and deoxygenated forms, respectively, via Autodock Tools [14]. With the same program, we also prepared GEM as a ligand. Then, using Autodock vina, [15] we docked GEM onto both structures within a searching box that would encompass the whole protein to look for potential binding sites.

### 2.4. Absorption Spectrum Analysis of HbS

UV-vis absorption spectra of HbS in PBS buffer were recorded using a Cary 60 Bio UV-visible spectrophotometer (Varian, Inc., Mulgrave, Australia). All measurements were performed at room temperature in 1 cm × 1 cm cuvettes.

### 2.5. In Vivo Drug Diffusion Study

HbS solution was prepared as described above. IR-780 iodide dye (Sigma Aldrich) was diluted in DMSO and mixed with HbS solution by vortexing for 30 s. For the HbS treatment group, a 1:1 (v/v) mixture of HbS solution with IR-780 dye solution was used. For the control group, a 1:1 (v/v) mixture of HbS solution with saline was used. A total of 100 μL was injected intratumorally in each tumor after the tumor had developed for 21 days as described below. Imaging was performed at 10 min, 30 min, and 1 h by IVIS live animal imaging machine.

### 2.6. In Vivo Tumor Efficacy Study

Animal procedures were performed according to protocols approved by the IACUC at the University of North Texas Health Science Center, Texas, USA. A subcutaneous xenograft tumor model was used to test in vivo drug and HbS synergistic efficacy. Four-to-six-week-old female athymic mice were donated from ENVIGO. Mice were allowed to acclimate for 1 week before experiments. Mice were injected with 1 × 10^6^ BxPC3 pancreatic cancer cells mixed 1:1 with Matrigel (BD Bioscience, San Jose, CA, USA) in a total volume of 0.2 mL. This was implanted to the subcutaneous area of the right flank of the nude mouse. Tumors developed for 1 week, then were randomized into groups by determining the tumor size using vernier caliper so that the starting average size was equal across treatment groups. Treatment commenced on day 7 with weekly 0.100 mL intratumoral injections. For the treatment experiment, mice (*n* = 7/group) bearing subcutaneous BXPC3 tumors were injected with saline, HbS (20 mg/mL) alone, GEM (50 mg/kg body weight) alone, and a combination of HbS and GEM (50 mg/kg body weight) intratumorally. Tumor sizes were measured at least twice a week for 27 days. When the tumor reached the final time point, the mouse was euthanized by CO_2_ asphyxiation. Tumor volume (mm^3^) was calculated as (length × width^2^)/2.

## 3. Results

### 3.1. In Silico Interaction Analysis of HbS and GEM

**Figure 1:**
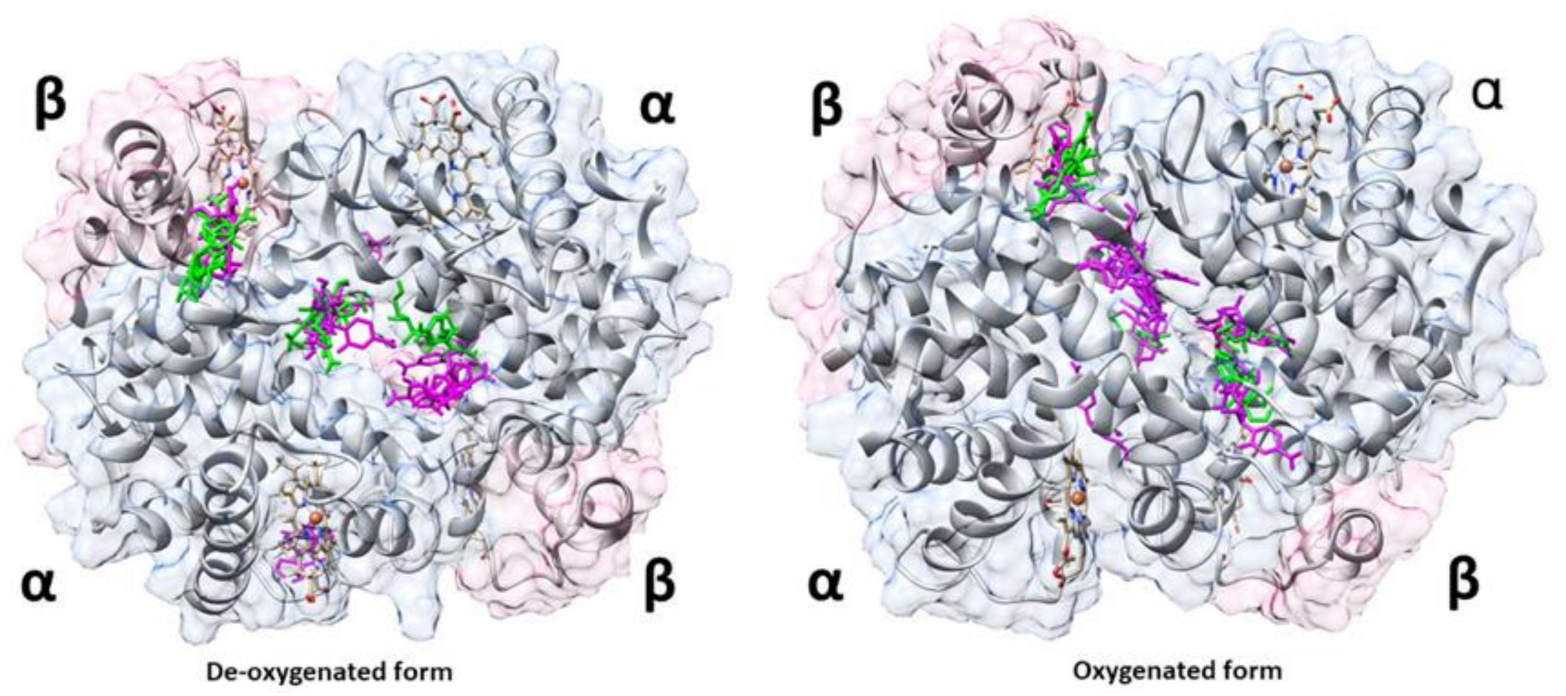
In silico molecular docking poses of gemcitabine on hemoglobin S tetramer in deoxygenated (**left**) and oxygenated (**right**) forms. In the deoxygenated form (**left**), the green poses are poses with binding energy of −6.0 to −6.4 kcal/mol and the pink poses are those with binding energies of −5.7 to −5.9 kcal/mol. In the oxygenated form (**right**), the green poses are poses with binding energy of −6.5 to −6.9 kcal/mol and the pink poses are those with binding energies of −6.0 to −6.4 kcal/mol. The best poses in both forms are those between the α and β chains. Figures were prepared with UCSF Chimera [16].

In order to determine if there is an interaction between HbS and GEM molecules, we performed in silico docking of GEM on HbS. There was a significant interaction between GEM and multiple areas on the HbS subunits, although the highest affinities were predicted for binding poses between the α and β chains (Figure 1). This interaction was modeled in both the oxygenated conformation and deoxygenated conformation of HbS. It was found that the poses of GEM on the deoxygenated form of HbS had lower affinities compared to the oxygenated form. This implies that GEM can be loaded on HbS while in an oxygenated state. Then, when HbS is in a hypoxic condition such as the tumor microenvironment, not only will it start to polymerize, but it will also offload any GEM that is bound to the protein. This is an important finding in this proposed treatment as this essentially demonstrates delivery of a potent drug at the target site. This ensures reduced off target effects of the drug. It has not escaped our attention that this offloading of drug from the HbS during a conformational change could also serve a role in loading other small molecules on HbS and systemically delivering chemotherapy to other tumor sites.

### 3.2. Characterization of Polymerization of Hemoglobin S under Hypoxia

**Figure 2:**
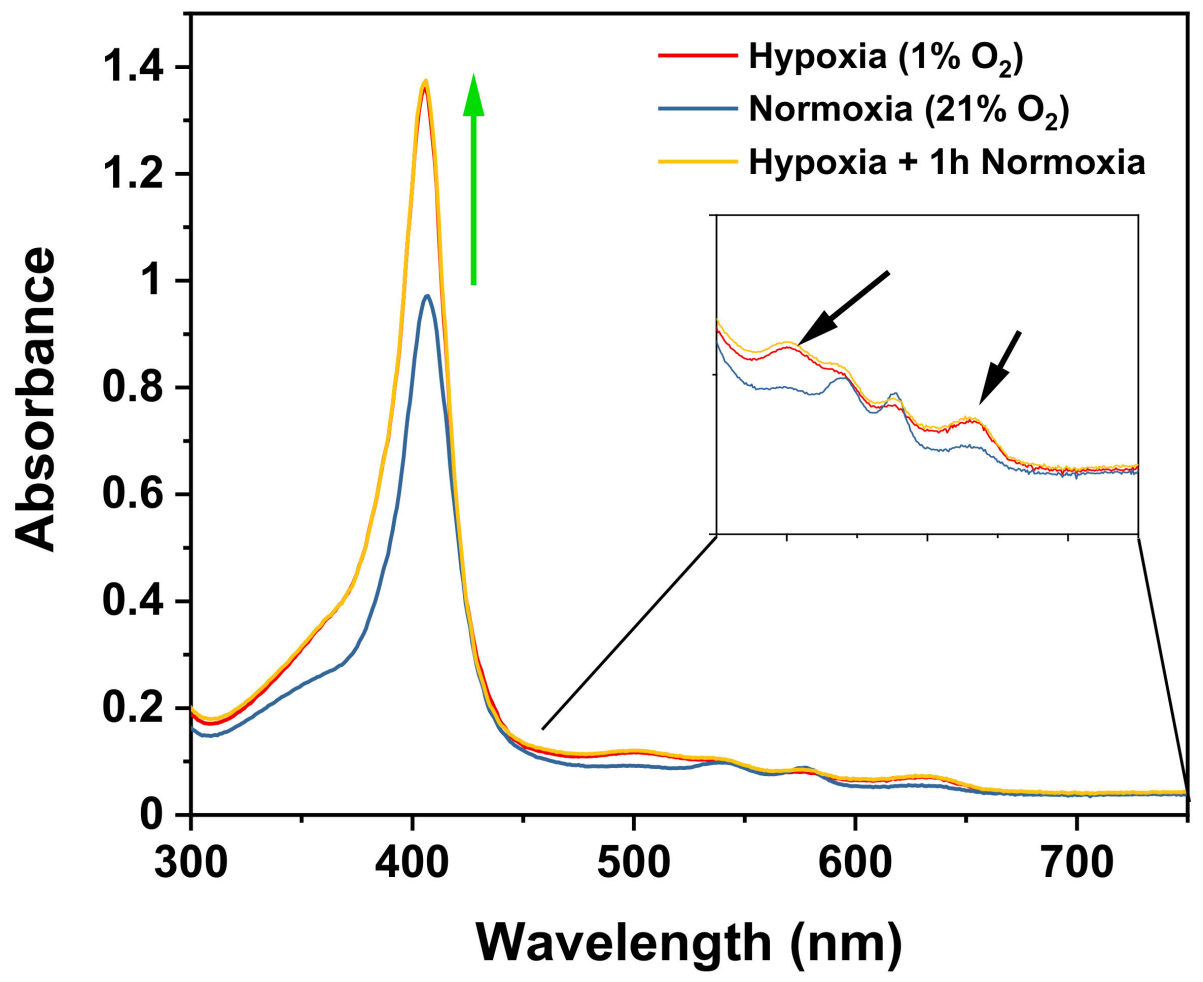
UV-vis absorption spectra of HbS protein after overnight treatment under hypoxia (1% oxygen), normoxia (21% oxygen), and after exposing the hypoxia samples to 21% oxygen (normoxia condition) at 37 °C for 1 h.

It was suspected that the structural changes of HbS due to polymerization under hypoxic conditions might be reflected in the form of a variation in absorption [17]. Therefore, the absorption spectrum of the HbS protein was recorded and compared under hypoxia and normoxia conditions. The reason that a spectral change occurs is that chromophores residing within chemical/biochemical molecules have varying interactions with the environment depending on their conformation. When a change in conformation occurs within a molecule, the chromophores become more shielded or less shielded from solvent molecules. The outcome is a change in the spectral properties of the chromophores and absorption characteristics observed. The comparison of the HbS absorption spectra under hypoxia (1% oxygen), normoxia (21% oxygen), and hypoxia after 21% oxygen (normoxia condition) exposure is presented in Figure 2. The intensity of the absorption transition near 400 nm significantly increases with hypoxia (green arrow). Additionally, there are new features in the absorption spectrum under hypoxia near 550 nm and 650 nm. Black arrows on Figure 2 insert show the areas where the changes occurred. Next, after the hypoxia treatment, HbS was exposed to 21% oxygen (normoxia condition, 1 h at 37 °C) to determine if the observed differences in absorption spectra were permanent conformational changes or reversible. This re-exposure to oxygen did not revert back to the original absorption spectrum seen under oxygenated conditions (normoxia), suggesting the conformation change from hypoxia was permanent (Figure 2).

### 3.3. In Vivo Drug Diffusion Tumor Model

An IR dye was used as a surrogate molecule for GEM to determine the effect HbS polymerization has on the ability of drugs to diffuse throughout the tumor. The IR dye was chosen based on its small molecular weight and ability to easily detect a fluorescent signal during live animal imaging.

**Figure 3:**
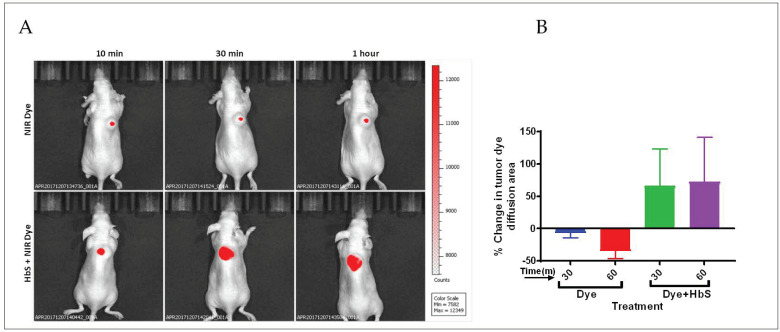
In vivo distribution of NIR dye (IR-780-I) in nude mice bearing xenografted BxPC3 pancreatic tumor at 10 min, 30 min, and 1 h after intratumoral injection (**A**). Fluorescence quantification of dye distribution in tumors at 30 min and 1 h (**B**).

The tumors treated with HbS trended toward having a more ubiquitous distribution of dye throughout the tumor after injection at 10 min, 30 min, and 1 h. This supports the notion that HbS polymerization disrupts the tumor microenvironment and can improve diffusion of small molecules throughout the tumor (Figure 3).

### 3.4. In Vivo Efficacy in Pancreatic Tumor Model

**Figure 4:**
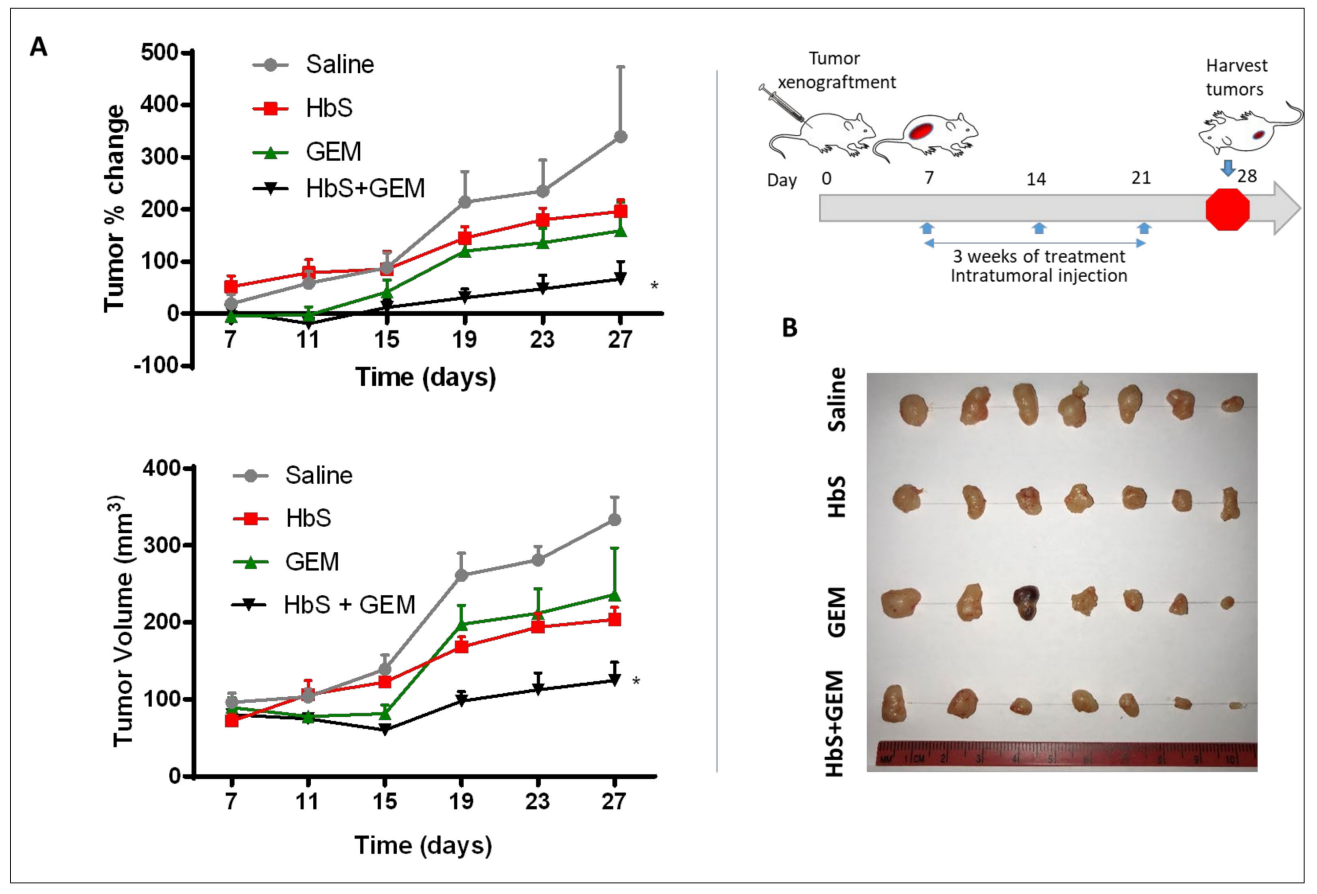
In vivo antitumor efficacy of GEM and HbS combination. Tumor-growth inhibition in mice treated with saline, HbS, GEM, and HbS + GEM by intratumoral injection (n = 7). Tumor percentage change and tumor volume (**A**). Data given as mean ± standard deviation (n = 7). (**B**) Photograph of tumors for the four groups of saline, HbS, GEM, and HbS + GEM. * *p* < 0.05; Abbreviations: GEM, gemcitabine; HbS, hemoglobin S.

A subcutaneous flank model of pancreatic cancer was used to study the effect of the HbS + GEM formulation. In Figure 4, mice treated with HbS + GEM resulted in the smallest tumor size throughout the study and at the final point. Interestingly, there was a trend toward a decreased tumor size in the group treated only with HbS compared to the control group. This may indicate that there is some degree of benefit in using solely HbS on the tumor microenvironment. The precise mechanism cannot be elucidated from the current study although a possible explanation is through decreasing interstitial tumor pressure. It has been shown in a number of studies that decreasing interstitial tumor pressure allows a better therapeutic response [6]. Alternatively, it is possible the HbS polymerization in the tumor causes direct physical disruption of the tumor cell membranes with a resulting decrease in tumor size. This would be similar in mechanism to the pathophysiology that occurs in patients with sickle cell anemia, where HbS sickles and causes disruption to red blood cell membranes. One main difference between this proposed therapy mechanism and sickle cell anemia sickling is that with the intratumoral injection, the vast majority of the sickling will occur inside the local tumor leaving healthy tissue intact. Another point that supports the notion that the HbS may have an impact on the tumor independently of the chemotherapy cargo is that the growth rate of the tumors from days 15–19 saw steeper growth curves in the groups where HbS was not present. It is possible that during this time the tumor is most responsive to changes in intratumoral pressure as mentioned above.

## 4. Discussion

There are some shortcomings with traditionally developed nanoparticle drug delivery systems due to the complicated chemical synthesis strategies required for manufacturing. Additionally, the drug carrier itself often does not act on the tumor microenvironment and functions solely as a delivery vessel for chemotherapy. Further, there is often a limitation of biocompatibility which can potentially lead to a decreased drug accumulation and a subsequent limited tumor response, as the immune system functions to clear out the foreign particles. Finally, the concept of exploiting pH as a chemotherapy release trigger mechanism has been studied by others in nanotechnology drug delivery platforms that include hydrogels [18,19,20,21], as well as others. While this has worked in preclinical models, only incremental progress has been made translating this to clinical success. Little has been investigated on utilizing a mechanism of simultaneously exploiting both the acidic and hypoxic tumor environment for drug delivery.

In this proof-of-concept manuscript we attempted to address these problems of nanoparticle drug carriers mentioned above. The HbS + GEM complex we have developed takes inspiration from the pathological hemoglobin molecule, HbS, which is known to polymerize in humans with sickle cell disease in conditions that are hypoxic and acidic. The simplicity of manufacturing this complex helped mitigate the time-consuming chemical synthesis process and allowed a rapid validation with in vitro and in vivo studies, as well as could lead to easing of the scaling up process required for clinical trials in the future. The simple design of this complex also allows for the possible creation of other hemoglobin–drug combination systems that may be useful in other cancers beyond pancreatic cancer, which was experimentally tested in this study. Importantly, the design of this system in which both the drug carrier itself and the drug have a specific antitumor purpose is a critical component. The HbS molecule naturally polymerizes in the same tumor microenvironment that supports tumor cell growth and evasion of chemotherapy. The development of a carrier that can physically disrupt the tumor microenvironment can add synergistic tumor toxicity to the cargo chemotherapy. Additionally, because the HbS differs only by one amino acid from the ubiquitously found natural hemoglobin molecule, we would expect some degree of immune privilege status, helping it to evade the immune system. Additional studies are needed to determine the toxicity of this system, but it is anticipated that the toxicity will be mitigated in healthy areas of the body that are appropriately oxygenated and at a physiologic pH.

This study highlights the potential of a novel bioinspired method of using the self-polymerizing ability of HbS together with GEM to improve local delivery in a complex tumor microenvironment. We have designed this therapy to be simply produced and locally delivered intratumorally through the use of endoscopic ultrasound. In vivo intratumoral distribution revealed that HbS improved the diffusion of small molecules in the tumor microenvironment. Importantly, the delivery of HbS and GEM acted together in reducing tumor size. Ultimately, this approach could possibly further reduce tumor burden in patients with locally advanced pancreatic cancer and potentially lengthen survival.

## Data Availability

The data presented in this study are available on request from the corresponding author.
